# Imaging Frontside and Backside Attack in Radical Ion–Molecule Reactive Scattering

**DOI:** 10.1021/acs.jpca.3c02856

**Published:** 2023-06-24

**Authors:** Atilay Ayasli, Arnab Khan, Tim Michaelsen, Thomas Gstir, Milan Ončák, Roland Wester

**Affiliations:** Institut für Ionenphysik und Angewandte Physik, Universität Innsbruck, Technikerstrasse 25/3, 6020 Innsbruck, Austria

## Abstract

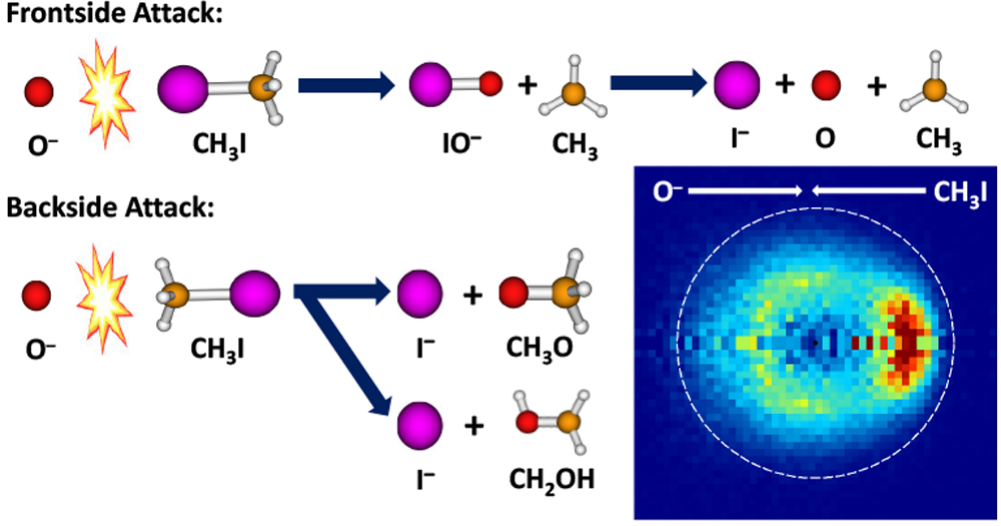

We report on the reactive scattering of methyl iodide, CH_3_I, with atomic oxygen anions O^*–*^. This radical ion–molecule reaction can produce different ionic products depending on the angle of attack of the nucleophile O^*–*^ on the target molecule. We present results on the backside and frontside attack of O^*–*^ on CH_3_I, which can lead to I^*–*^ and IO^*–*^ products, respectively. We combine crossed-beam velocity map imaging with quantum chemical calculations to unravel the chemical reaction dynamics. Energy-dependent scattering experiments in the range of 0.3–2.0 eV relative collision energy revealed that three different reaction pathways can lead to I^*–*^ products, making it the predominant observed product. Backside attack occurs via a hydrogen-bonded complex with observed indirect, forward, and sideways scattered iodide products. Halide abstraction via frontside attack produces IO^*–*^, which mainly shows isotropic and backward scattered products at low energies. IO^*–*^ is observed to dissociate further to I^*–*^ + O at a certain energy threshold and favors more direct dynamics at higher collision energies.

## Introduction

The study of gas-phase reaction dynamics is a field of research that provides deep insight into the atomistic details of chemical reactions.^1−3^ An important part of this are reactions involving ions,^4−7^ which are widely present from the interstellar medium^8,9^ to the human body,^10^ and can also be found in different phases of matter.^11,12^ However, unlike liquid and solid phases, in gas-phase ion–molecule reactions, the interaction potential and associated dynamics can be treated at a single-molecule level. Therefore, they provide ideal exemplary systems to combine experiments and theoretical modeling of complicated chemical reactions and ion–molecule interaction potentials.^13−16^ Yet, the related dynamics are quite complex. As a result, depending on the energetics of the reaction, these scattering processes often lead to different product pathways, even in simple systems.^3,17,18^ Furthermore, these pathways also depend on the relative orientation of the reactants during the collision.

In the case of bimolecular nucleophilic substitution (S_N_2) reactions, the potential energy surface (PES) generally involves a reaction barrier that separates pre- and postreaction complexes.^19^ Most elementarily, an S_N_2 reaction has the form X^–^ + CH_3_Y → CH_3_X + Y^–^, where X^–^ denotes the nucleophile and Y^–^ denotes the leaving group. The nucleophilic attack results in the formation of a C–X bond and the cleavage of the C–Y bond, with the concerted formation of a Y^–^ leaving ion. In the course of this reaction, a Walden inversion, i.e., an inversion of the CH_3_ group, takes place. Different mechanisms have been identified for this reaction, notably the direct rebound mechanism that is initiated by backside attack and the frontside attack, during which the nucleophile attacks on the side of the leaving group and no inversion of the CH_3_ group occurs.^5^ The latter mechanism is generally sterically hindered and has a higher energy barrier than the more common backside attack. Alternatively, a stripping mechanism may take place when the nucleophile approaches CH_3_Y at a larger impact parameter and strips away the CH_3_ moiety. Additionally, several indirect mechanisms are possible, which can proceed via one or a combinations of processes such as the formation of a hydrogen-bonded prereaction complex, roundabout, barrier recrossing, etc.^5^ Besides nucleophilic substitution, also other reaction channels become accessible with increased collision energy, such as proton-transfer with the creation of CH_2_Y^–^ product ions, or halide abstraction with the formation of XY^–^ ions.^20,21^

The type of nucleophile significantly affects the reaction dynamics.^22^ Previously, our group studied F^–^, Cl^–^, OH^–^, and CN^–^ reactions with CH_3_I and CH_3_Cl for a range of collision energies between 0.3 and 3.0 eV.^14,15,20,22−24^ It has been observed that the nucleophile strongly influences the entrance channel complexes and barrier heights. Especially, the PESs of the F^–^ and OH^–^ reactions with CH_3_I differ significantly from the characteristic double-well structure for a typical S_N_2 reaction found, for example, in Cl^–^ + CH_3_I.^5^ For F^–^/OH^–^ reactions, in addition to the F^–^···CH_3_I ion–dipole complex there is a chance to create F^–^···HCH_2_I (or OH^–^···HCH_2_I) like hydrogen-bonded complexes. All of these effects make the F^–^/OH^–^ induced S_N_2 reaction more indirect than Cl^–^ induced S_N_2. These observations are generally connected to the differences in the structure of the PES. For very large reaction exothermicities, the barrier height is strongly decreased and may eventually vanish,^25^ an effect that correlates with the ionization potential of the attacking nucleophile.^25,26^

Radical anions, such as O^–^, are known to be highly reactive. They can be used to synthesize active organic intermediates, act as oxygen donor in biological oxidation processes, and are relevant for the oxidation of solid surfaces and in radiolytic chemistry.^27^ Oxygen radical anions are suspected to act as a source of damage to DNA or RNA strands in biological media, where they are formed via dissociative electron attachment to water molecules.^28^ The gas-phase dynamics of O^–^ reactions have been studied for collisions with a range of molecules, such as H_2_, D_2_, HF, NH_3_, H_2_O, and C_2_H_2_.^29−35^ Specifically, the reaction like O^–^ + CH_4_ → OH^–^ + CH_3_ was studied as the prototype to understand hydrogen abstraction mechanisms related to the methane oxidation processes in combustion chemistry.^36^

Here, we report on the dynamics of the O^–^ + CH_3_I radical anion–molecule reaction. Measurements of the collision-energy product branching ratio show that the formation of I^–^ product anions dominates the reaction. The O^–^ anion has an electron detachment threshold of 1.42 eV, which is much less than that of F^–^ (3.56 eV) or Cl^–^ (3.72 eV) and still smaller than that of OH^–^ (1.85 eV).^26^ Therefore, the O^–^ is a better electron donor than the previously studied closed-shell nucleophiles, and it is interesting to compare the results for the O^–^ reaction with those of the reactions of F^–^, Cl^–^, and OH^–^. The reactive channels of O^–^ leading to proton transfer and combined hydrogen/proton transfer have been reported recently.^37^ The experimental results are supported by quantum chemical calculations of the minimum-energy pathways and transition states.

## Experimental Methods

The experiment was performed using a crossed beam setup equipped with a velocity map imaging (VMI)^38^ spectrometer. A detailed description of the experimental setup has been described previously.^15,37,39,40^ In short, O^–^ ions are produced by a plasma discharge source. For the present study, we have used a 1–2% mixture of N_2_O with Ar as a precursor for the creation of O^–^. First, the O^–^ ion packet is guided by a combination of Wiley–McLaren^41^ type electrodes, lenses, and electrostatic deflectors before it gets thermalized to room temperature by helium buffer gas in an octupole radio frequency ion trap. After being trapped, the ion-packet is decelerated to a desired energy and crossed with the neutral CH_3_I beam, seeded in helium, at the center of the VMI-spectrometer. The resulting product ions are then imaged with a time and position-sensitive detector. In this way, we measure the detector position and time-of-flight of each ionic reaction product event-by-event and then transform these into the three-dimensional velocity vector of the ion after the reaction.

## Results and Discussion

We have carried out scattering measurements at eight relative collision energies between 0.3 and 2.0 eV. Several different product channels are energetically accessible in this range:
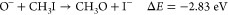
1
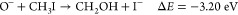
2
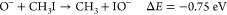
3
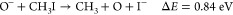
4
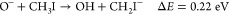
5
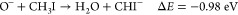
6All listed product ions have been detected in the present experiment. In addition, there is a possible O^–^ + CH_3_I → OH^–^ + CH_2_I channel. This channel has been mentioned in previous studies,^27^ but it could not be detected here, because trace amounts of OH^–^ or ^17^O^–^ in the reactant ion beam mask this product in our experiment.

*Ab initio* calculations were carried out at the CCSD(T)/MP2 level of theory to obtain the energetics of the different reaction pathways in reactions 1–6. All structures were optimized at the MP2 level of theory using the aug-cc-pVTZ-PP basis set for iodine and aug-cc-pVTZ for other atoms. Wave function stability with respect to relaxing various constraints was tested prior to every calculation, and if an instability appeared, then the wave function was stabilized. The obtained structures were recalculated at a single point at the CCSD(T) level of theory with the same basis set. The CCSD(T) energies were corrected for zero-point energies at the MP2 level. See the Supporting Information for benchmarking calculations and Cartesian coordinates of all optimized structures. The Gaussian software was used for all calculations.^42^

**Figure 1:**
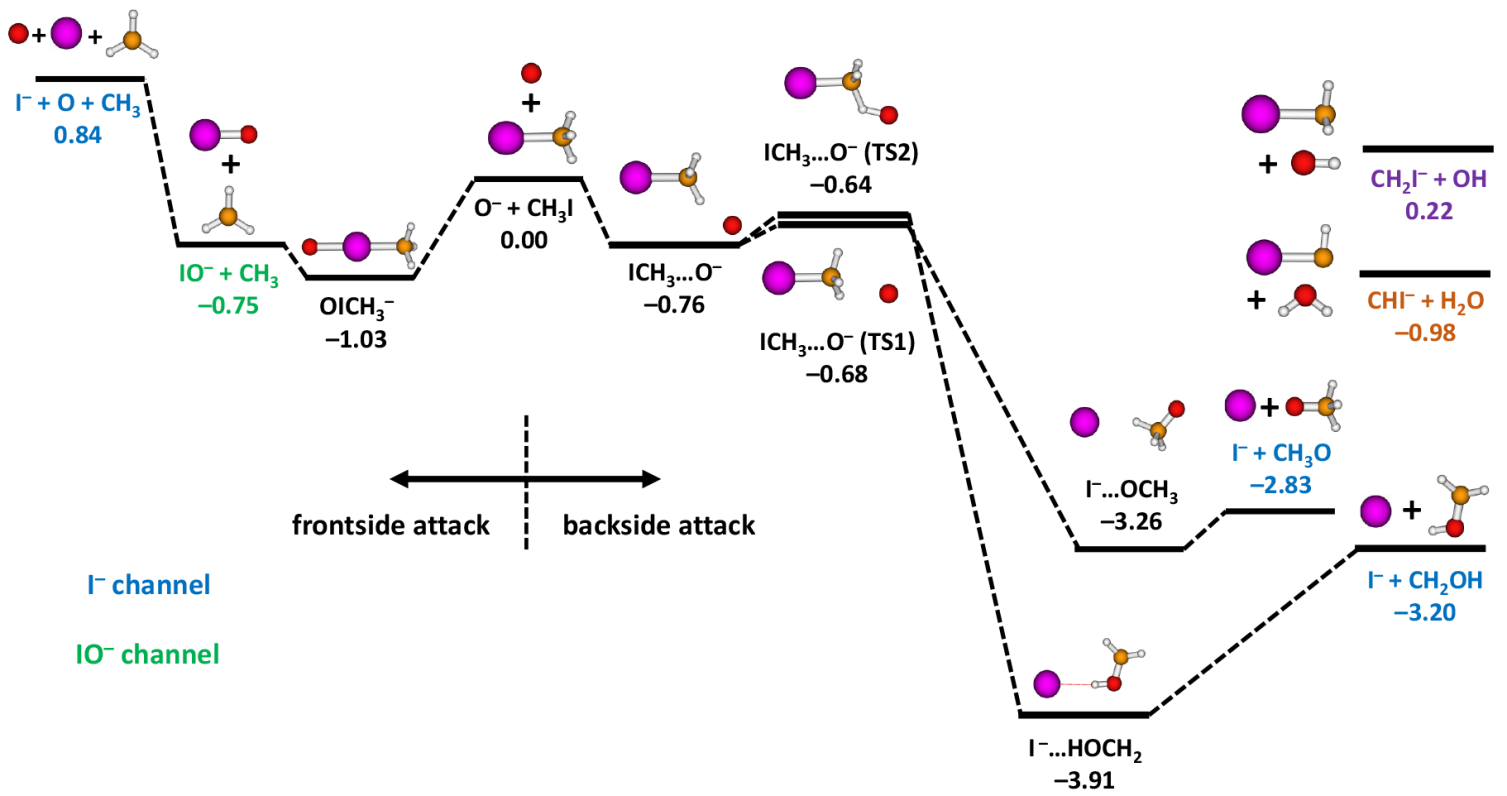
Calculated minimum-energy pathways of reaction channels 1–4 at the CCSD(T)/aug-cc-pVTZ(-PP)//MP2/aug-cc-pVTZ(-PP) level. Optimized geometrical structures are also shown for each stationary point on the PES, and TS1 and TS2 mark the two transition states (energy unit is eV). In addition, the previously studied product channels of reaction pathways 5 and 6 are shown.^37^ Color code: oxygen, red; carbon, brown; hydrogen, white; iodine, violet.

The resulting energy levels for the different reaction pathways are listed in Figure 1. The calculated reaction pathways show that three different pathways can lead to the I^–^ product ions. Nucleophilic substitution, reaction 1, follows a pathway across TS1 and via a postreaction complex to the products CH_3_O + I^–^ with Δ*E* = −2.83 eV. Alternatively, oxygen insertion reaction 2, leads to I^–^ and the formation of CH_2_OH as the neutral coproduct (Δ*E* = −3.20 eV). This pathway contains the same prereaction complex as reaction 1, but then the O^–^ ion interacts more strongly with one of the hydrogens of CH_3_I and reaches TS2. As the reaction proceeds, a bond-isomerization happens and oxygen is inserted into CH_3_I, which leads to a postreaction complex of I^–^··· HOCH_2_ shape. The third channel that leads to the formation of I^–^ proceeds via a barrierless reaction forming IO^–^, which can subsequently dissociate into I^–^ + O.

**Figure 2:**
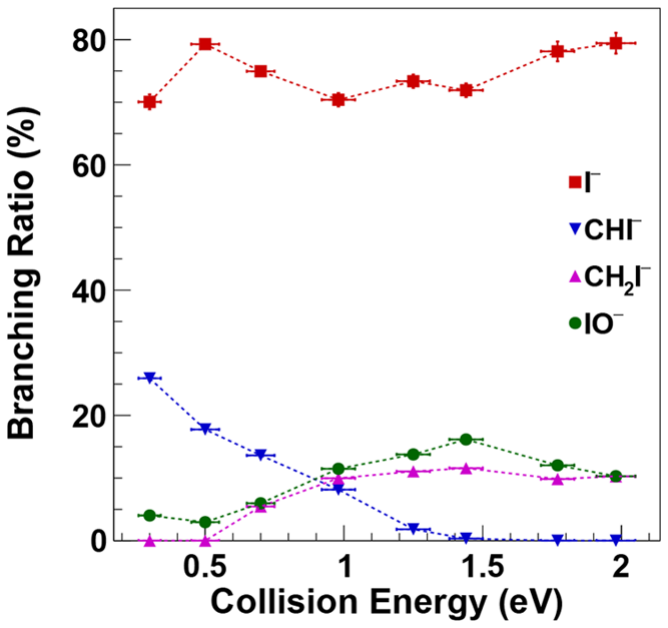
Experimental branching ratio of the product masses obtained from the reactive scattering of the O^–^ + CH_3_I as a function of collision energies. The branching is calculated by fitting Gaussians and finding the area under each time-of-flight distribution. Error in the *y*-axis shows the error obtained during the Gaussian fitting, while the error along the *x*-axis represents the uncertainty in determining the collision energy.

The branching ratios of the different observed product ions I^–^, IO^–^, CH_2_I^–^, and CHI^–^ are shown in Figure 2 as a function of collision energy. The dominant product ion at all collision energies is I^–^ with a branching ratio of about 70%–80% throughout the investigated collision energy range. In addition, also IO^–^ and CHI^–^ are formed at low collision energies, while CH_2_I^–^ appears only above about 1 eV collision energy. The reaction pathways that form CH_2_I^–^ via proton transfer (reaction 5) and CHI^–^ via combined hydrogen/proton transfer (reaction 6) have already been discussed previously.^37^ The reaction pathway leading to IO^–^ is exothermic with Δ*E* = −0.75 eV, in agreement with observation, and also barrierless (see Figure 1). However, as illustrated in Figure 2, its branching ratio is quite low at the lowest collision energies and increases only slightly at intermediate collision energies. This may be explained by the higher exothermicity for reactions 1 and 2, compared to reaction 3, or by steric hindrance because the halide abstraction leading to IO^–^ has to proceed via a frontside attack. When the collision energy is increased, IO^–^ products can break apart and lead to the channel shown in reaction 4. This channel is endothermic by 0.84 eV with respect to the entrance channel asymptote. Evidence of IO^–^ fragmentation is depicted in Figure 2. There, a clear decrease in the IO^–^ branching ratio above 1.5 eV collision energy is accompanied by a corresponding increase in the I^–^ signal. Additional evidence for this fragmentation emerges in the IO^–^ product velocity images discussed further below.

**Figure 3:**
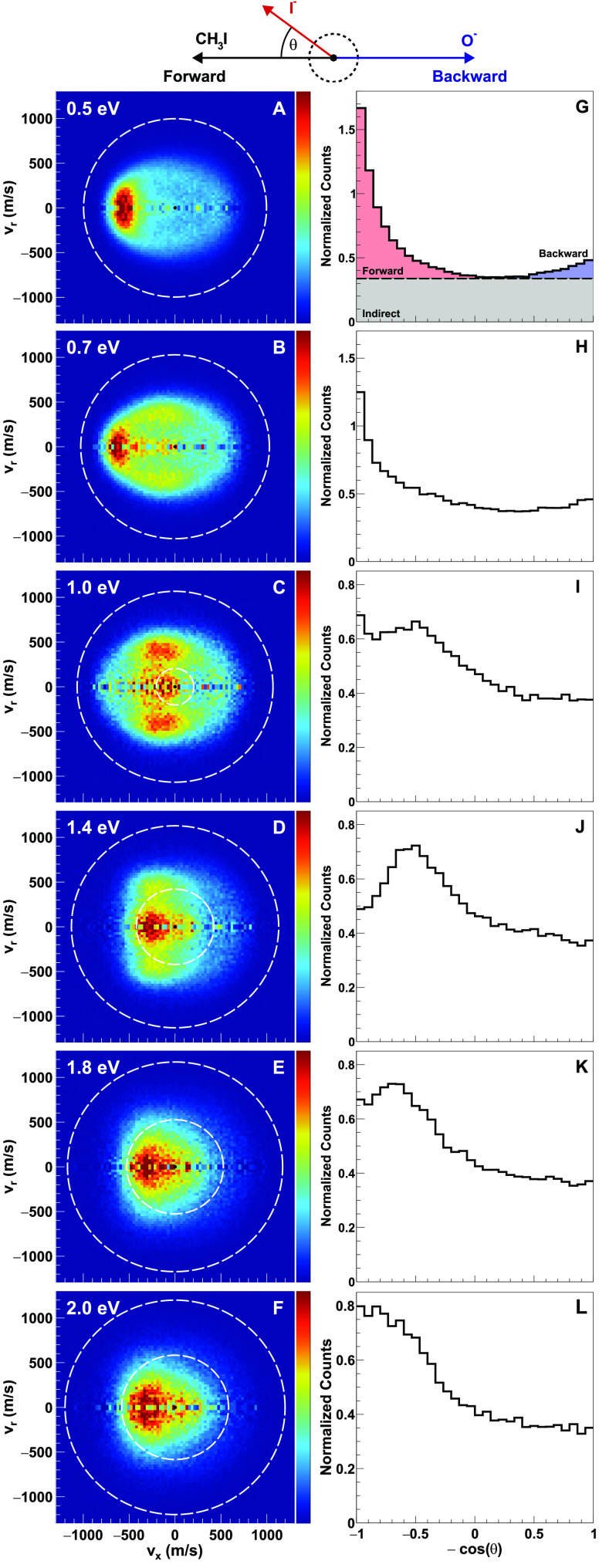
(A–F) Measured center-of-mass frame velocity images of the I^–^ product from the O^–^ + CH_3_I reactive scattering at different collision energies. The Newton diagram at the top depicts the relative orientations of the velocity vectors of the reactants and the I^–^ product ions. The white dotted circle with a bigger radius represents the kinematic cutoff for the O^–^ + CH_3_I → I^–^ + CH_3_O pathway, while the circle with a smaller radius stands for the kinematic cutoff for the O^–^ + CH_3_I → I^–^ + O + CH_3_ pathway. (G–L) Velocity integrated angular distributions for I^–^. Different dynamical features can be seen, which are fingerprints of different reaction mechanisms, such as forward scattering, backward scattering, and indirect. (G) A representative illustration of analysis processes for how we calculate the contribution of flux in each of the above-mentioned channels.

More detailed information about the reaction dynamics is obtained from the velocity distributions of the I^–^ and IO^–^ product ions. The I^–^ velocity distributions in the center-of-mass reference frame are shown in Figure 3A–F. The velocity vectors of the two reactant beams in the center-of-mass frame and the direction of the product ions are defined in the Newton diagram at the top of Figure 3. The white-colored dashed outermost circle in the images indicates the kinematic cutoff for the S_N_2 channel (reaction 1), i.e., the maximum available product kinetic energy for each collision energy given by conservation of energy and momentum. The inner circle in Figure 3C–F represents the kinematic cutoff for the three-body breakup channel (reaction 4). At the lowest collision energies (Figure 3A,B), the velocity and angular distribution of iodide anions show a strong forward scattered contribution. This observation signifies large impact parameter collisions and can be usually attributed to the forward stripping mechanism.^5,43,44^ This forward-dominated behavior is also evident in the angular distributions of the I^–^ product, shown in Figure 3G,H. As the relative collision energy increases, the maximum impact parameter for capture behind the centrifugal barrier decreases. Here, the contributions of isotropic indirect scattering and sideways stripping become more pronounced (Figure 3C,D). The slow I^–^ products near the center of the velocity distributions are the signature of a transient reaction complex that leads to high internal excitation of the neutral coproduct(s).

Throughout all investigated collision energies, an indirect mechanism dominates in the I^–^ velocity distributions. This manifests itself as a uniform I^–^ distribution without an angular dependence on the velocity distributions. It is also visible in the angular distribution plots of the I^–^ ions as isotropic distributions (Figure 3G–L). With further increase in collision energy (around 1.8 eV, see Figure 3E), sideways scattering vanishes and the I^–^ product distribution becomes predominantly forward scattered. This could be explained by the dissociation of IO^–^ product ions, as further discussed below. For all of the collision energies considered here, backward scattered products contribute only weakly to the overall differential scattering image. This implies the absence of the direct rebound mechanism in the O^–^ + CH_3_I reaction system, in contrast to reactions of F^–^ with CH_3_Cl or Cl^–^ or CN^–^ with CH_3_I.^23,24,45^

**Figure 4:**
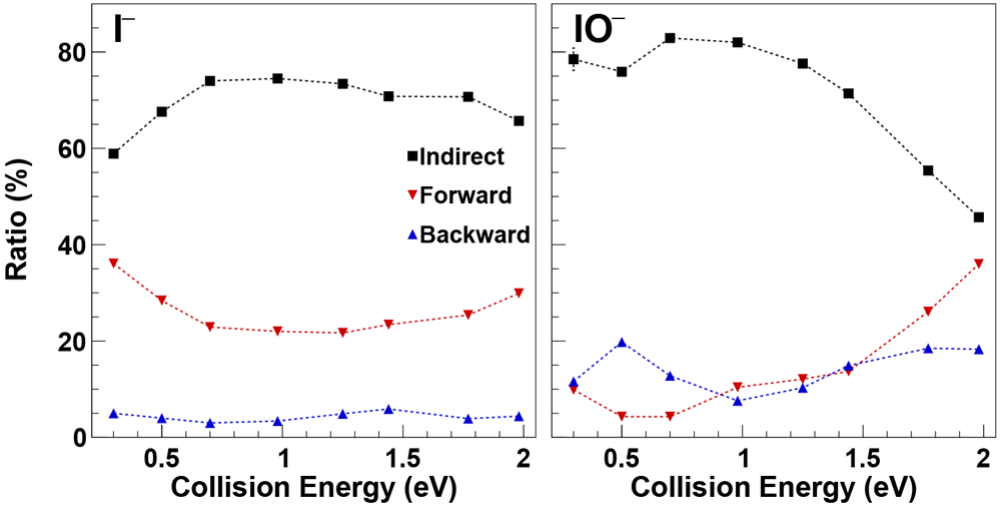
(left panel) Normalized experimental branching ratio of I^–^ ion flux in forward, backward, and indirect scattering as a function of collision energy. (right panel) Normalized experimental branching ratio of IO^–^ ions in forward, backward, and indirect scattering as a function of collision energy.

To get a quantitative idea of the relative contributions of the different channels and their energy-dependent behavior, we divide the I^–^ angular distributions into three different parts and integrate the signal in each of them. The procedure is illustrated in Figure 3G, which shows the isotropic contribution in gray, forward scattered flux in red, and backscattered signal in blue. The integrated results for all collision energies are shown in Figure 4 (left panel). We find that the indirect contribution dominates for all energies with a ratio of between 58% and 75% of the total I^–^ flux. Forward scattering along with sideways stripping accounts for about 20–35% of the total I^–^ flux, while backward scattering events comprise only about 4–5% of the events. This gives more quantitative evidence that the direct rebound mechanism via a colinear backside attack of the O^–^ nucleophile has only a minor probability. This analysis gives only an upper limit for the indirect mechanism and lower limits for forward and backward scattering. Nonetheless, the relative trend of the different channels with changing collision energy should still be reliable. We have also addressed this in our recent publication on the separation of S_N_2 and E2 processes.^3^

The observed high probability of the indirect channel may be attributed to both the S_N_2 and oxygen-insertion pathways (reactions 1 and 2). According to our calculation, both pathways proceed via the same prereaction complex and have only a very small energy difference between the transition-state complexes. This prereaction complex shows a hydrogen-bonded oxygen anion instead of a colinear complex with *C*_3*v*_ symmetry. Such hydrogen-bonded complexes have been observed previously for F^–^ and OH^–^ collisions with CH_3_I, where they were found to lead to a large fraction of indirect dynamics.^15,22,46^ However, when we compare the isotropic distribution of the present reaction with the F^–^ and OH^–^ cases,^15,22^ we notice that for F^–^ and OH^–^ the isotropic distributions are localized at small absolute velocities. For the present reaction, however, the isotropic part of the scattering image has a larger outer velocity radius (Figure 3A–F). This suggests that for the O^–^ radical anion reaction, the transient complex is too short-lived to allow for the efficient distribution of translational energy into rovibrational modes of the product.

**Figure 5:**
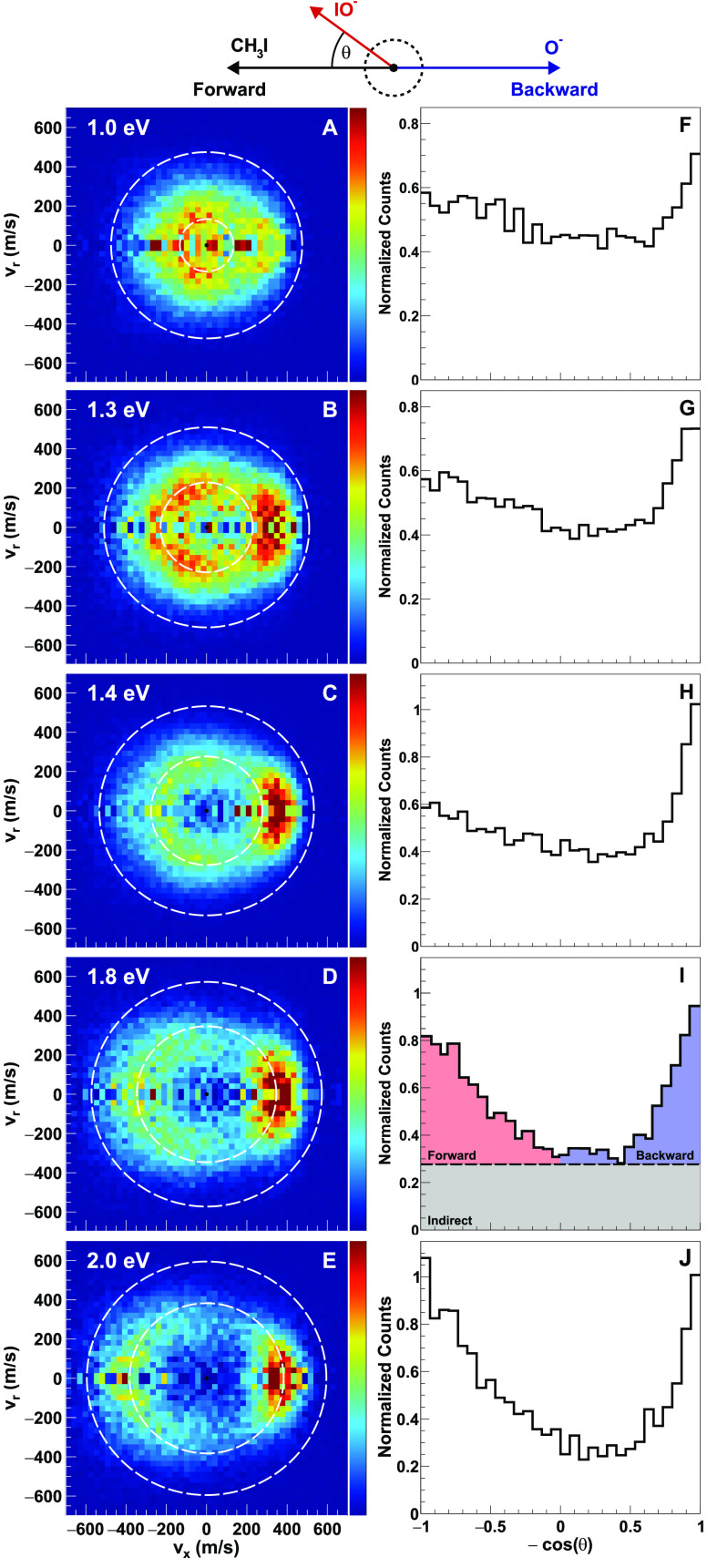
(A–E) Center-of-mass frame velocity images of the IO^–^ ions from the O^–^ + CH_3_I reactive scattering at different collision energies. The Newton diagram at the top illustrates the relative orientation of the velocity vectors of the reactants and the IO^–^ product ions. White dotted bigger circle represents the kinematic cutoff for the O^–^ + CH_3_I → IO^–^ + CH_3_ pathway, while the smaller circle depicts the kinematic cutoff for the O^–^ + CH_3_I → I^–^ + O + CH_3_ pathway. (F–J) Velocity integrated angular distributions for IO^–^ products. (I) A representative illustration of analysis processes for how we calculate the contribution of flux in forward scattering, backward scattering, and the indirect channel.

In Figure 5A–E, we show the measured velocity and angle differential cross sections of the IO^–^ product ions. The corresponding angular distributions are plotted in Figure 5F–J. The images show that for the lowest collision energies (0.7–1.0 eV) the distributions are mostly isotropic with a preference for backward scattering. As *E*_col_ increases, the IO^–^ tends to break apart into I^–^ + O. This becomes evident at 1.3 eV collision energy (Figure 5B), where we observe the onset of a ring-type structure in the velocity distribution with a central hole. The radius of this structure increases with increasing collision energy (Figure 5B–E), in good agreement with the calculated energy threshold for IO^–^ dissociation depicted by the inner dashed circle. It is interesting to note that qualitatively somewhat similar scattering dynamics have been extracted from crossed-beam measurements of the neutral O atom reaction with CH_3_I, albeit at a lower collision energy.^47^

We have extracted the fraction of indirect, forward, and backward scattered flux from the IO^–^ angular distributions, similar to the procedure for I^–^ (see Figure 5I and Figure 4, right panel). As the collision energy is increased, the indirect IO^–^ flux decreases and the forward and backward scattered part increases. The isotropic part of the velocity distribution is a fingerprint for a complex-mediated indirect mechanism. Its reduction with collision energy is an indication for the decrease of the lifetime of the respective reaction complex, which in this case is assumed to be a frontside O^–^···ICH_3_ complex. Such a decrease has been observed previously for the F^–^ + CH_3_I system.^48^ The frontside complex CH_3_···I···O^–^ also has almost the same binding energy (1.03 eV with respect to reactants as that of CH_3_···I···F^–^ (0.99 eV) and CH_3_···I···OH^–^ (1.04 eV) complexes and also similar geometrical structures.^48^

At the highest studied collision energies, the IO^–^ velocity distributions resemble nearly forward–backward symmetric images. Forward scattering even becomes stronger than backward scattering (Figure 4, right panel). This energy-dependent variation between forward and backward scattering distributions may point toward the lifetime of the complex being close to its rotational period.^49^ Similar collision energy-dependent oscillatory behavior in the angular distributions has been seen before for [FCH_3_I]^−^ and IO···HO^–^ complexes.^50,51^

## Conclusion

In summary, we have studied the O^–^ + CH_3_I reaction as a model radical anion–molecule system. Unlike many previously studied systems (F^–^/Cl^–^/OH^–^/CN^–^ + CH_3_I, F^–^ + CH_3_Cl), our electronic structure calculations of the O^–^ + CH_3_I energetics show a rich variety of different pathways for different angles of attack of the O^–^ nucleophile. By analyzing the differential scattering cross section for the I^–^ and IO^–^ product ions as a function of collision energy, we have identified different reaction mechanisms that happen via both backside attack and frontside attack. Combined with the energetics calculations we find that backside attack occurs via a hydrogen-bonded complex leading to indirect reaction dynamics as well as forward and sideways scattered products. Halide abstraction via frontside attack becomes visible through the IO^–^ formation channel, which is exothermic for the present reaction of radical oxygen ions, in contrast to the previously studied reactions of closed-shell nucleophiles. The IO^–^ product is observed to dissociate when the collision energy is increased above 1.3 eV, which adds a third pathway to the formation of I^–^ ions. We expect that these radical anion–molecule reactive scattering results will inspire further experimental and theoretical studies of the rich dynamics of such reactive systems.

## Data Availability

The underlying data for this study are available at DOI: 10.5281/zenodo.7995646.

## Supporting Information Available

Details of the electronic structure calculation (PDF)
