# Supplementary material for: Imaging Frontside and Backside Attack in Radical Ion–Molecule Reactive Scattering
Source: J Phys Chem A. 2023 Jun 24;127(26):5565–71. doi: 10.1021/acs.jpca.3c02856 (PMC10331732; doi:10.1021/acs.jpca.3c02856)
Supplement: Supplementary file 1 — jp3c02856_si_001.pdf [file jp3c02856_si_001.pdf]

Supporting Information

for

Imaging Frontside and Backside Attack in Radical Ion-Molecule Reactive Scattering

Atilay Ayasli, Arnab Khan, Tim Michaelsen, Thomas Gstir,
Milan Ončák, and Roland Wester*

Institut für Ionenphysik und Angewandte Physik, Universität Innsbruck, Technikerstraße 25/3, 6020 Innsbruck, Austria

Electronic mail: roland.wester@uibk.ac.at

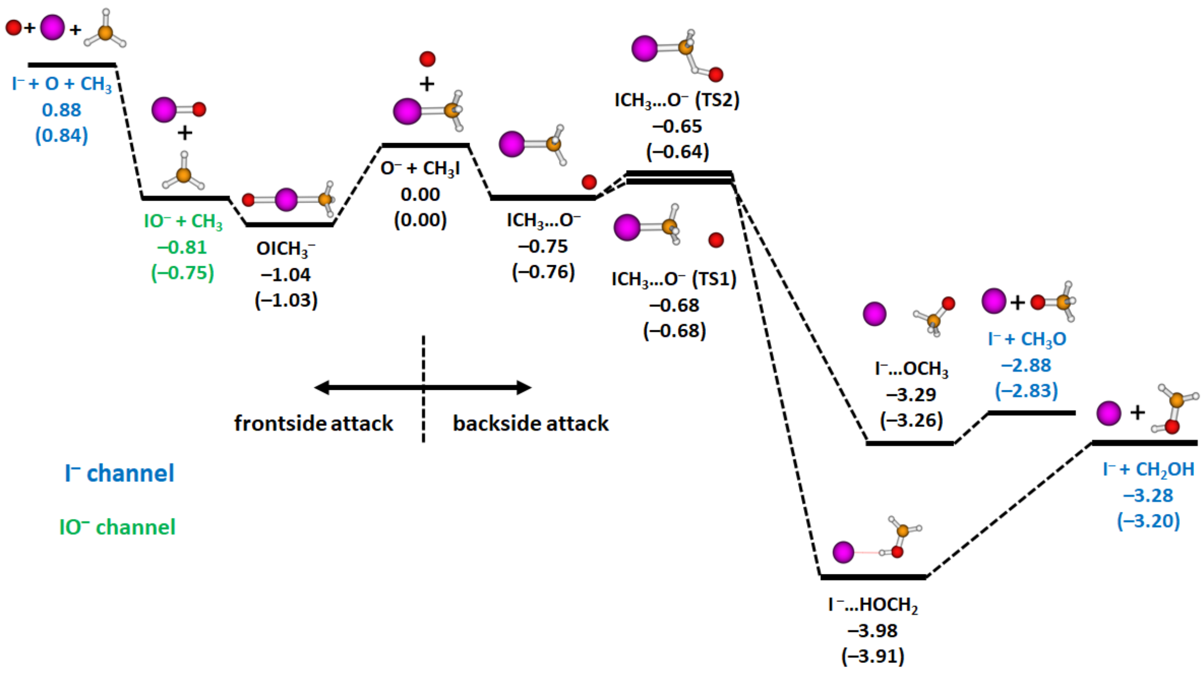

Figure S1 – Reaction profile of selected pathways of the O^- + CH_3I reaction as calculated at the CCSD(T)/aug-cc-pVQZ(-PP)//MP2/aug-cc-pVTZ(-PP) level. The values obtained at the CCSD(T)/aug-cc-pVTZ(-PP)//MP2/aug-cc-pVTZ(-PP) level are given in parenthesis. Color code: oxygen – red, carbon – brown, hydrogen – white, iodine – violet.

Cartesian coordinates (in Å) and zero-point corrected energies (in Hartree) of various ions and molecules optimized at the MP2/aug-cc-pVTZ(-PP) level

CH_2OH
E = -114.828514
o -0.670344 -0.125770 0.021786
h 1.228740 -0.883135 0.109462
c 0.684839 0.027454 -0.066718
h 1.113350 0.987789 0.174110
h -1.088370 0.736781 -0.057552

CH_3
E = -39.708081
C -0.000000 0.000000 0.000063
H 0.000000 0.930926 0.537191
H -0.000000 -0.930926 0.537191
H 0.000000 0.000000 -1.074762

CH_3I
E = -334.659616
I -0.000001 0.324146 0.000000
C -0.000001 -1.799162 0.000000
H -1.031804 -2.128261 0.000000
H 0.515943 -2.128254 0.893559
H 0.515943 -2.128254 -0.893559

$I^-..OCH_3$
E = -409.786357
c 0.421134 -2.722895 0.000000
i 0.000000 0.998153 0.000000
h 1.033682 -2.746264 0.905326
h 1.033682 -2.746264 -0.905326
h -0.076155 -1.726772 0.000000
o -0.564752 -3.668180 0.000000

$OICH_3^-$
E = -409.707224
c 0.009982 -2.198847 -0.000000
i 0.000000 0.034394 0.000000
h -1.018008 -2.560036 -0.000000
h 0.523403 -2.559651 0.890603
h 0.523403 -2.559651 -0.890603
o -0.011086 2.381190 0.000000

CH_3O
E = -114.806347
O 0.009327 0.794471 -0.000000
C 0.009327 -0.582085 0.000000
H 0.460922 -0.999622 0.902107
H 0.460922 -0.999622 -0.902107
H -1.052421 -0.864014 0.000000

$I^-…HOCH_2$
E = -409.820956
o 2.448216 -0.698201 0.008532
h 4.024227 0.603458 0.192503
c 2.976876 0.544141 -0.059387
h 2.312384 1.384452 0.080125
h 1.462017 -0.600230 -0.022945
i -0.853691 0.017605 0.000724

$ICH_3…O^-$
E = -409.698207
i -0.729693 -0.100642 -0.000000
c 1.107032 0.999160 -0.000002
o 3.489156 -0.520202 0.000001
h 1.976236 0.267767 -0.000221
h 1.070943 1.616616 -0.892867
h 1.071132 1.616279 0.893098

I^-
E = -294.965831
I 0.000000 0.000000 0.000000

O^-
E = -75.011329
o 0.000000 0.000000 0.000000

OI^-
E = -369.996287
O 0.000000 0.000000 -1.670574
I 0.000000 0.000000 0.252162

O
E = -74.959294
o 0.000000 0.000000 0.000000

$ICH_3…O^-$ (TS1)
E = -409.694565
o -3.822123 -0.167944 -0.001162
c -1.326268 0.303274 0.002107
i 0.819814 -0.028994 -0.000218
h -1.688668 0.049904 0.986710
h -1.463550 1.348839 -0.228727
h -1.763350 -0.338149 -0.749800

$ICH_3…O^-$ (TS2)
E = -409.688720
o 3.254494 -0.365650 0.000000
c 1.322970 0.620476 0.000000
h 2.125092 -0.428479 0.000000

h 1.425142 1.211569 0.900843
h 1.425142 1.211568 -0.900843
i -0.734890 -0.052685 0.000000
